# Association of PON1 gene polymorphisms, paraoxonase 1 gene expression and activity in Egyptian children and adolescents with sickle cell disease

**DOI:** 10.1186/s12887-026-06597-w

**Published:** 2026-03-21

**Authors:** Diana Hanna, Amr I.Risha, Mai M.Eldaly, Hosnia Ragab, Maha Mahmoud Hamed Sakr, Alaa Nafea, Maha Abdelaziz Noah, Hala Mohammed Amin Abdelmoneim, Sara F. Saadawy

**Affiliations:** 1 Pediatric Department, Faculty of Medicine, Zagazig University, Zagazig, Egypt; 2 Medical Biochemistry Department, Faculty of Medicine, Zagazig University, Zagazig, 44523 Egypt; 3 Public Health and Community Medicine Department, Faculty of Medicine, Zagazig University, Zagazig, Egypt; 4 Clinical Pathology Department, Faculty of Medicine, Zagazig University, Zagazig, Egypt

**Keywords:** Sickle cell disease, PON-1, Polymorphism, Expression

## Abstract

**Introduction:**

Sickle cell disease (SCD) is defined by chronic oxidative stress, hemolysis, vaso-occlusive crisis (VOC), endothelial injury, and end-organ damage. Paraoxonase1 (PON-1) serves a significant function as an anti-inflammatory and antioxidant agent. This study investigates PON1c.55L > M and PON1c.192Q > R polymorphism frequency in Egyptian children and adolescents with SCD, as well as the impact of these polymorphisms on PON-1 gene expression and enzymatic activity.

**Methods:**

The study recruited 70 SCD children in addition to 70 healthy matched participants as controls. Laboratory parameters, PON-1 gene molecular analysis, PON-1 gene expression, and enzymatic activity were investigated.

**Results:**

PON-1 activity and expression were substantially reduced in patients with SCD than in controls (*P* < 0.001). We detected higher allelic frequencies of PON1c.192R (52.9% in SCD cases and 25.7% in controls). For *PON1*c.192Q > R, compared with the genotype QQ, we found genotype QR, and RR were significantly associated with increased SCD risk.

Alternatively, the PON1c.55L > M variant allele had no significant association with SCD risk. Furthermore, SCD cases with the homozygote variant PON1c.192 RR, PON1c.55 MM genotypes exhibited decreased PON1 activity relative to controls possessing corresponding genotypes. SCD patients with the RR genotype in SCD exhibited notable reductions in RBC count, MCH, and MCHC, alongside substantial rises in WBCs, neutrophil count, LDH, ferritin, creatinine, cystatin C, and BUN levels. Additionally, substantial correlations were identified between the MM genotype variant and decreased RBC counts, alongside a notable increase in WBC count, serum ferritin, and cystatin C levels. A positive correlation between PON-1 activity and hematocrit (*r* = 0.26; *P* < 0.05) was identified in patients with SCD. Conversely, negative correlations were found with WBCs (*r* = -0.47; *P* < 0.001), creatinine (*r* = − 29; *P* < 0.05), cystatin C (*r* = − 37; *P* < 0.001) and ferritin levels (*r* = − 0.34; *P* < 0.05).

**Conclusion:**

The PON1c.55L > M and PON 1c.192Q > R gene polymorphisms in SCD cases influenced the PON-1 gene expression and activity. SCD cases with the variant PON1c.192 RR, PON1c.55 LL and MM genotypes exhibited downregulated PON1 activity. This reduction was correlated with elevated hemolysis and inflammation markers, ferritin levels, creatinine, cystatin C, and BUN levels, indicating a potential link between decreased PON1 activity and renal and cardiovascular dysfunction incidence.

**Supplementary Information:**

The online version contains supplementary material available at 10.1186/s12887-026-06597-w.

## Introduction

SCD is a hereditary monogenic disorder characterized by a point mutation that leads to the substitution of glutamic acid with valine at the sixth position of the hemoglobin globin chain. SCD includes HbS/β-thalassemia, HbSC disease, and homozygous SCD [1, 2]. HbS polymerizes (in the deoxygenated state), altering the erythrocyte's rheology. This polymerization of hemoglobin is considered the first incident in a pathophysiological cascade of hemolysis, VOC, endothelial injury, chronic oxidative stress, and end-organ damage such as left ventricular diastolic heart dysfunction, pulmonary hypertension, sudden death, stroke, hepatic and renal functions [3–5].

PON genes comprise three members, PON-3, PON-2, and PON-1 (located at chromosome 7q21–23), with a reported variability of PON activity as a result of these gene polymorphisms [6–8].

PON1 is an aryldialkyl phosphatase glycoprotein primarily synthesized in the liver, released into circulation, and delivered to target tissues via its association with apoA-I of HDL-C [9]. PON-1 substantially contributes to low-density lipoprotein (LDL) oxidation. Oxidized LDL is associated with a high risk of endothelial injury and inflammation [10, 11]. In addition, PON-1 can hydrolyze phospholipid hydroperoxides, which play a significant role in detoxifying oxidative stress intermediates and may elucidate the enzyme's anti-inflammatory as well as antioxidant properties [7, 12].

Two polymorphisms were recognized in the coding region of the PON1 gene: the first involves the substitution of glutamine for arginine at position 192 (PON1c.192Q > R), and the second involves the substitution of leucine with methionine at position 55 (PON1c.55L > M) [6]. The variability in PON-1 genotypic frequency distribution, expression, and enzymatic activity was observed across various populations and diseases [13].

Evaluating PON1 activity in SCD is essential due to its role in oxidative stress regulation and vascular homeostasis, both of which are known to be disrupted in SCD pathophysiology [14].

This study was conducted to examine *PON1*c.55L > M and *PON1*c.192Q > R polymorphism frequency in Egyptian children and adolescents with SCD, the influence of these polymorphisms on PON-1 gene expression and enzymatic activity, and the correlation between laboratory and clinical parameters as well as PON1 activity in cases with SCD. To our knowledge, this is among the first studies to examine the association between PON1 polymorphisms, expression, and enzymatic activity in a pediatric SCD population from Egypt, a population with unique genetic and environmental characteristics. These findings may offer ethnic-specific insights into oxidative stress and vascular risk in children with SCD.

## Material and methods

The study was conducted at the Pediatric Hematology outpatient clinic, Department of Pediatrics, and Medical Biochemistry Department, Faculty of Medicine, Zagazig University. The Faculty of Medicine's Institutional Review Board (Zagazig University, IRB No. ZU-IRB #58/17-Jan-2024) approved the study protocol. Written informed consent was secured from all legal guardians for the utilization of samples and clinical data. Consent was obtained using a specific form in accordance with the Declaration of Helsinki 1964 and its following amendments.

A case–control study involved 70 SCD children and adolescents (HbSC genotype, HbSβ0/β + thalassemia, and HbSS) aged ≤ 18 years. Enrolled SCD patients were required to be in a steady state, defined as being at least three months post-blood transfusion and at least fourteen days following any SCD complications, such as hospitalization due to stable Hb baseline level, VOC, or acute chest syndrome (ACS) To ensure the accuracy and consistency of biochemical measurements, a stable baseline hemoglobin level was established for all participants as Sickle cell disease is characterized by frequent fluctuations in hemoglobin concentration due to acute events such as vaso-occlusive crises, infections, hemolysis, or recent blood transfusions. These events can significantly influence oxidative stress markers and (PON1) enzyme activity, potentially confounding the assessment of gene expression and genotype–phenotype relationships. This ensured that observed differences in PON1 expression and activity were more likely due to genetic factors rather than transient changes in disease status. This approach enhances the reliability of the data and the validity of comparisons both within the patient group and between patients and healthy controls. In addition to a stable baseline hemoglobin (Hb) level, participant must have been on a consistent dose of hydroxyurea therapy (mg/kg) for a minimum of three months prior to enrollment. A reference group of 70 healthy children, matched for age and sex, was included; all exhibited HbAA profiles. Patients receiving chronic blood transfusion therapy were excluded. However, patients who had received occasional transfusions during acute events were included. Also, those with chronic comorbid conditions, liver or renal impairment, central nervous system disorders, malignancy, chronic infections or inflammatory disorders, and dyslipidemia were excluded from the study.

We extracted medical and medication data from the patient's medical records. The collected data focused on family history (refers to the presence of sickle cell disease (SCD) or sickle cell trait in first-degree relatives (parents or siblings), as reported by the patients or their guardians), disease duration (the period from the first clinically documented SCD-related event to the time of patient recruitment for the study), frequency and severity of VOCs and ACS, blood transfusion history, chelation history, Hydroxyurea use, dosage, and compliance with therapy.

### Laboratory investigations

CBC (Complete blood count)was performed by (Sysmex KX-21). Renal and liver function tests were conducted utilizing the Dimension E.S. chemical autoanalyzer. Serum ferritin levels were assessed utilizing a human ELISA kit (Thermo Fisher-Vilnius-Lithuania). Lipid and iron profiles were determined using a Modular 48 Analytics P-800 automated analyzer (Roche Diagnostics Corporation-Indianapolis-IN-USA).

### Blood sampling

A venous blood sample of 5 ml was obtained. Following a 15-min coagulation period, serum separation was done through centrifugation for 10 min (at 4000 × g). Prior to measuring PON activity, serum samples were kept at −80°C. A separate collection of whole blood samples was obtained and transferred to EDTA tubes. They were then promptly kept at −80°C until they were needed for genotyping and DNA extraction.

### Paraoxonase activity

Senti et al. [15] and Agachan et al. [16] provided the information needed to calculate PON-1 activity. P-nitrophenol is produced when paraoxon is hydrolyzed enzymatically. The generation rate of this compound was measured spectrophotometrically using absorbance measurements at 405 nm over 10 min at 37 °C.

### Real-time quantitative polymerase chain reaction and RNA extraction

Total RNA was extracted from 500 μL serum samples using TRIzol reagent (Invitrogen, USA). Determination of extracted RNA concentration was done by assessing the absorbance at 260 nm (A260), while RNA purity was evaluated utilizing absorbance value ratio at 260 nm and 280 nm (A260/A280). A260/A280 ratio indicative of pure RNA ranges from 1.9 to 2.1. Total RNA was reverse-transcribed utilizing the QuantiTect Rev. Transcription Kit (Qiagen) following the manufacturer's guidelines. PON1 mRNA's relative expression was assessed in a 25 μl reaction mixture containing 50 ng cDNA, 1 μl of 10 pM of each primer (forward and reverse), and 12.5 μl SYBR Green master mix (TOPreal SYBR Green, Enzynomics, Korea), utilizing the Stratagene Mx3005P qPCR System, with β-actin serving as an internal standard control. The cycling conditions included an initial activation step at 95 °C for 10 min, followed by 40 cycles consisting of 30 s at 95 °C, 60 s at 60 °C, and 30 s at 70 °C. Gene expression was quantified using the 2^−ΔΔCT^ method [17]. The primer sequences utilized were: PON1 (forward 5′ -GTATTTGGGTTTAGCGTGGTCG-3′; reverse 5′ -GACATACTTGCCATCGGGTGA-3′) and β-actin (forward 5′ -CTTCCTTCCTGGGCATG-3′; reverse 5′ -GTCTTTGCGGATGTCCAC-3′).

### Extraction of DNA and subsequent genotyping analysis

Genomic DNA was extracted per the manufacturer's instructions utilizing the G-spinTM Total DNA Extraction Mini Kit (iNtRON Biotechnology-Inc.-Republic of Korea-Catalogue No. 17046). The PON1c.55L > M (rs854560) and PON1c.192Q > R (rs662) polymorphism genotyping were genotyped utilizing TaqMan SNP Genotyping Assays (Applied Biosystems-Thermo Fisher Scientific-Foster City-CA-USA; SNP Assay ID: C__2548962_20, C__2259750_20). Reactions were conducted in a StratageneMx3005P Real-Time PCR System according to the specified protocol: thermal cycling conditions included an initial step for 10 min at 95 °C, followed by 40 cycles for 15 s at 95 °C and finally for 1 min at 60°C.

### Statistical analysis

All analyses were conducted utilizing the V 26.0 of IBM SPSS Statistics (Armonk-New York-USA). Categorical data were expressed as numbers and percentages, while quantitative data were presented as range, SD, and mean. For analysis of categorical variables (e.g., Transfusion, Iron Chelation, Genotypes), we used the Chi-square test (X2). For continuous variables (e.g., Disease Duration, Age), we used ANOVA when comparing across three genotype groups. For non-normally distributed data, non-parametric tests such as the Kruskal-Wallis test were applied. Quantitative data were investigated for normality utilizing the Kolmogorov-Smirnov test. Variables with a normal distribution (among two groups) were compared utilizing the Student t-test, whereas the Mann-Whitney U test was utilized for variables with non-normal distribution. The Pearson`s correlation coefficient assessed the correlation between two quantitative variables. *P* ≤ 0.05 was considered statistically significant. To account for multiple comparisons and reduce the risk of Type I error, Bonferroni correction was applied where appropriate, particularly in genotype-based subgroup analyses. Adjusted p-values were reported and interpreted accordingly. Figures were generated using GraphPad Prism v9.0

## Results

### Demographic and laboratory parameters

This study comprised 70 patients diagnosed with SCD (30 (42.86%) females and 40 (57.14%) males), with a mean age of 8.9 ± 3.6 years and 64 ± 3.6 months mean disease duration. The control group consisted of 70 individuals (38 (54.29%) males, 32 (45.71%) females) with a mean age of 9.7 ± 2.1 years. Among the patients, 42 (60%) had HbSS, four (5.7%) with HbSC genotype, and 24 (34.3%) with Sickle β-thalassemia. In the past 12 months, 34 patients (48.6%) experienced < 1 VOC, whereas 36 patients (51.4%) experienced > 2 VOCs. In the past 12 months, 34 SCD patients (48.6%) experienced < 1 ACS, while > 2 ACS were documented in 51.4% of patients. In total, 34 patients (48.6%) underwent iron chelation therapy using deferasirox film-coated tablets, with full compliance observed among all participants. PON1 expression and activity were markedly diminished in patients with SCD relative to controls as demonstrated in Table 1.

**Table 1:** Comparison between SCD patients and controls regarding laboratory parameters

Laboratory parameters	SCD (***n*** = 70)	Control group (***n*** = 70)	***P*** value
	Mean ± SD	Mean ± SD	
Hemolysis markers			
RBC (10^12^/mL)	3.28 ± 0.04	4.5 ± 0.4	< 0.001**
Hemoglobin (g/dL)	8.5 ± 0.9	12.8 ± 1.2	< 0.001**
Hematocrit (%)	26.7 ± 4.5	38.5 ± 3.7	< 0.001**
MCV (fL)	85.9 ± 4.6	79.00 ± 5.7	< 0.001**
MCH (ρg)	29.3 ± 4.3	27.2 ± 3.4	< 0.001**
MCHC (g/dL)	52.00 ± 02. 1	33.2 ± 2.3	< 0.001**
Reticulocyte count (%)	8.7 ± 4.6	0.9 ± 0.8	< 0.001**
Total bilirubin (mg/dL)	3.00 ± 0.7	0.7 ± 0.2	< 0.001**
Direct bilirubin (mg/dL)	0.6 ± 0.1	0.29 ± 0.07	< 0.001**
Indirect bilirubin (mg/dL)	2.4 ± 0.2	0.4 ± 0.2	< 0.001**
LDH (U/L)	531.00 ± 200.00	391.00 ± 89.00	< 0.001**
Leukocytes			
WBC (10^9^/L)	8.4 ± 3.4	6.5 ± 0.8	< 0.001**
Neutrophils (10^9^/L)	61.00 ± 7.3	48.7 ± 6.0	< 0.001**
Monocytes (10^9^/L)	6.8 ± 1.8	4.00 ± 0.9	< 0.001**
Lymphocytes (10^9^/L)	51.3 ± 6.4	29.9 ± 5.6	< 0.001**
Platelets			
Platelet count (10^3^/mL)	375.00 ± 83.5	294.00 ± 34.00	< 0.001**
Lipid profile			
Total Cholesterol (mg/dL)	128.9 ± 18.00	133.00 ± 28.00	0.24
HDL-C (mg/dL)	35.7 ± 4.7	47.0 ± 6.3	< 0.001**
LDL-C (mg/dL)	72.4 ± 12.3	75.1 ± 12.2	0.19
Triglycerides (mg/d)	118.8 ± 21.00	79.8 ± 16.00	< 0.001**
Iron metabolism			
Iron (mcg/dl)	125.32 ± 18.00	78.5 ± 12.7	< 0.001**
Renal profile			
Creatinine	2.4 ± 3.6	0.7 ± 0.10	< 0.001**
Urea	38.4 ± 20.3	26.7 ± 5.6	< 0.001**
Cystatin C	7.3 ± 7.0	0.9 ± 0.3	< 0.001**
BUN	3.2 ± 0.8	1.9 ± 0.5	< 0.001**
Albumin/creatinine ratio	3.00 ± 1.9	0.9 ± 0.1	< 0.001**
Hepatic profile			
AST (U/L)	44.6 ± 12.00	29.00 ± 4.7	< 0.001**
ALT (U/L)	38.5 ± 7.9	25.7 ± 3.8	< 0.001**
Inflammatory biomarkers			
Ferritin (ng/dL)	268.4 ± 204.00	39.2 ± 7.2	< 0.001**
ESR	17.4 ± 11.9	5.4 ± 3.2	< 0.001**
CRP (mg/L)	3.9 ± 3.0	2.4 ± 2.00	< 0.001**
Paraoxonase1 activities Paraoxonase (U/mL)	73.83 ± 53.00	102.8 ± 49.3	< 0.001**
Paraoxonase1 expression	0.99 ± 0.1	1.00 ± 0.04	< 0.001**

*RBC* Red blood cells, *MCV* Mean cell volume, *MCH* Mean corpuscular hemoglobin, *MCHC* Mean corpuscular hemoglobin concentration, *LDH* Lactate dehydrogenase, *WBC* White blood cell, *HDL-C* High-density lipoprotein cholesterol, *LDL-C* Low-density lipoprotein cholesterol, *AST* Aspartate aminotransferase, *ALT* Alanine amino-transferase, *CRP* C-reactive protein, *BUN* Blood Urea Nitrogen, *ESR* Erythrocyte Sedimentation Rate

^**^indicate high significance at *p* < 0.001

### *PON1 c.192Q > R* and *PON1 c.55L > M* genotype and allele frequency

The selected SNP genotype distribution as well as their correlations with SCD risk depicted Table 2. SNP genotype frequencies among controls and cases aligned with HWE (*P*-value > 0.05) as the PON1 c.55L > M polymorphism in the control group was in Hardy–Weinberg equilibrium, a slight deviation was observed for c.192Q > R (*p* = 0.035) (supplementary 1). The additive model results demonstrated a strong connection between *PON1c.192Q* > *R* variant allele distribution and SCD risk. In the case of *PON1c.192Q* > *R*, genotypes QR and RR exhibited a substantial assosciation with elevated SCD risk compared to the QQ genotype.

**Table 2:** Comparison between SCD patients and controls regarding *PON1* c.192Q > R Genotype and allele frequency

Polymorphism	SCD		Control			*OR (95%CI)*
	**N**	**%**	**N**		**%**	
*PON1c.192Q > R*						
QQ	18	25.7	42	60		1 (reference)
QR	30	42.9	20	28.6		3.5 (1.4–8.37)*
RR	22	31.4	8	11.4		6.4 (2.19–19.4) *
Allele frequency						
Q	66	47.1	104	71.3		1 (reference)
R	74	52.9	36	25.7		3.24(1.9–5.54)*

*SCD* Sickle cell disease, *N* Patient number

The *PON1c.55L* > *M* variant allele did not demonstrate a substantial connection with the risk of SCD. In the case of *PON1c.55L* > *M*, genotypes LM and MM exhibited an insignificant relationship with SCD risk compared to genotype LL (Supplementary 2).

### PON1 activity and PON1 expression in different genotypes for* PON1 c.192Q* > *R *and* PON1 c.55L* > *M *polymorphisms among SCD

Analysis of PON1 activity based on genotypes revealed notable differences between control and SCD groups. SCD patients possessing the homozygote variant *PON1*c.192 RR genotype demonstrated a substantial reduction in PON1 activity relative to controls with the corresponding genotype (*p* < 0.001). The *PON1*c.55L > M polymorphism indicates that SCD patients possess the wild-type PON1c.55 LL genotype exhibited a notable reduction in PON1 activity relative to controls with the corresponding genotype (*p* = 0.01). Additionally, patients with the MM variant genotype exhibited a statistically significant reduction in PON1 activity relative to controls of the corresponding genotype (*p* < 0.001) (Table 3).

**Table 3:** PON1 activity and PON1 expression in different genotypes for *PON1* c.192Q > R and *PON1* c.55L > M polymorphisms among SCD patients and controls

Polymorphism	PON1 activity (U/mL))			PON1 expression (fold change)		
	SCD (***n*** = 70)	Controls (***n*** = 70)	***P*** value	SCD (***n*** = 70)	Control (***n*** = 70)	***P*** value
*PON1*c.192Q > R						
QQ	144.9 ± 39.4	123.8 ± 39.6	0.06	0.92 ± 0.33	1.01 ± 0.05	0.07
QR	70.4 ± 25.5	88.1 ± 44.8	0.08	0.96 ± 0.1	1.0 ± 0.001	0.14
RR	20.4 ± 5.7	29.5 ± 4.1	< 0.001**	0.88 ± 0.06	1.0 ± 0.001	< 0.001**
*PON1* c.55L > M						
LL	98.7 ± 59.00	136.4 ± 34.1	0.01*	0.95 ± 0.18	1.01 ± 0.05	0.07
LM	78.2 ± 22.00	86.00 ± 45.5	0.5	1.0 ± 0.07	1.0 ± 0.001	0.29
MM	22.4 ± 16.00	49.8 ± 16.9	< 0.001**	0.85 ± 0.05	1.03 ± 0.05	< 0.001**

*SCD* Sickle cell disease

Bold values indicate significance

Analysis of PON1 expression based on genotypes revealed notable differences between the control and SCD groups. The PON1c.192Q > R polymorphism is associated with substantially decreased PON1 expression in SCD cases with the RR genotype (*p* < 0.001). The PON1c.55L > M polymorphism shows that SCD patients with the MM variant genotype exhibit a substantial decline in PON1 expression (*p* < 0.001) (Table 3).

### *PON1c.192Q* > *R and PON1 c.55L* > *M *polymorphism and demographic and clinical variants

No substantial variations were found in basic characteristics, such as family history, sex, disease duration, and age, except for disease duration, which showed a significant association with the PON1c.192Q > R polymorphism in the RR genotype, indicating that a shorter duration correlates with increased severity. Patients with QQ genotypes exhibited a significantly increased likelihood of elevated HbSS, ACS < 1, and absence of chelation therapy. The observed elevated ferritin levels in some patients may reflect previous episodic transfusions or inflammation, rather than chronic transfusion therapy (Table 4). Alternatively, the *PON1*c.55L > M *variant* had no significant association with basic characteristics and clinical variants (supplementary 3).

**Table 4:** Association between PON1c.192Q > R polymorphism and demographic and clinical variants

Variant	*PON1*c.192Q > R						
	QQ		QR		RR		*P*
	*N* = 18	%	*N* = 30	%	*N* = 22	%	
Sex							0.9
Male	10	55.6	18	60	12	54.5	
Female	8	44.4	12	40	10	45.5	
Age (Χ ± SD)	9.5 ± 3.8		9.6 ± 3.3		7.4 ± 3.3		0.06
Duration (Χ ± SD)	7.8 ± 3.8		7.1 ± 3.4		4.3 ± 2.96*		0.003*
Family history							
-ve	4	22.2	12	40.0	4	18.2	0.7
+ ve	12	77.8	18	60.0	18	81.8	
Diagnosis							
HbSS	16	88.9	12	40.0	14	63.6	0.001**
HbSB	2	11.1	14	46.7	8	36.4	
HbSC	0	0.0	4	13.3	0	0.0	
VOC							
≤ 1	6	33.3	18	60	10	45.5	0.18
≥ 2	12	66.7	12	40	12	54.5	
ACS							
≤ 1	18	100	22	73.3	20	91.9	0.03*
≥ 2	0	0	8	26.7	2	9.1	
Transfusion							
No	4	22.2	12	40	8	36.4	0.4
Occasional	14	77.8	18	60	14	63.6	
Chelation							
No	18	100	24	80	16	72.7	0.02*
Yes	0	0	6	20	6	27.3	

### *PON1c.192Q* > *R and PON1 c.55L* > *M *polymorphism and laboratory variants

This study examined the relationship between laboratory parameters in SCD and PON1 polymorphisms. The RR genotype was linked to notable reductions in MCHC, RBC count, and MCH, alongside substantial rises in BUN, WBC count, LDH, neutrophil count, ferritin, cystatin C, and creatinine levels (Table 5). Regarding PON1 c.55L > M, significant associations were observed between the MM genotype and reduced RBC counts, alongside notable increases in WBC counts, ferritin, and cystatin C levels (Table 6).

**Table 5:** Association between *PON1*c.192Q > R polymorphism and laboratory variants

	*PON1*c.192Q > R ( Χ ± SD)			
	QQ (***n*** = 18)	QR (***n*** = 30)	RR (***n*** = 22)	***P*** value
RBCs	3.5 ± 0.5	3.3 ± 0.4	3.16 ± 0.3*	0.02*
Haemoglobin	8.6 ± 0.9	8.5 ± 0.3	8.3 ± 0.9	0.3
Hematocrit	20.7 ± 5.6	26.2 ± 3.5	26.1 ± 4.3	0.1
MCV	87.0 ± 5.1	86.0 ± 5.2	84.7 ± 3.0	0.2
MCH	30.3 ± 3.0	30.0 ± 3.7	26.3 ± 5.4*	0.001*
MCHC	32.6 ± 2.5	32.1 ± 2.0	30.9 ± 1.4*	0.02*
Reticulocyte count	7.6 ± 3.9	7.8 ± 3.7	10.1 ± 5.0	0.1
Total bilirubin	2.98 ± 0.3	3.1 ± 0.7	3.2 ± 0.8	0.14
Direct bilirubin	0.6 ± 0.1	0.63 ± 0.1	0.6 ± 0.1	0.4
Indirect bilirubin	2.4 ± 0.3	2.4 ± 0.8	2.6 ± 0.7	0.5
LDH	433.3 ± 179	469.8 ± 213	640 ± 176**	0.001**
WBC	6.3 ± 1.9	8.3 ± 3.5	10.3 ± 3.4**	0.001**
Neutrophils	59.0 ± 9.5	60.8 ± 7.5	66.3 ± 7.3	0.004*
Monocytes,	7.3 ± 1.0	6.9 ± 2.1	6.4 ± 1.2	0.28
Lymphocytes	53.3 ± 5.4	50.1 ± 6.5	51.3 ± 7.0	0.25
Platelet count	387.8 ± 109	375.2 ± 86.9	363.0 ± 50.0	0.6
Lipid profile				
Total Cholesterol	125.0 ± 18.2	128.6 ± 18.0	134.0 ± 19.6	0.2
HDL-C	35.0 ± 5.2	37.0 ± 3.8	35.1 ± 4.0	0.1
LDL-C	72.4 ± 15	71.0 ± 10.3	74.0 ± 12.0	0.59
Triglyceride	115.4 ± 26.0	116.3 ± 17.0	127.0 ± 21.0	0.16
Iron	119.4 ± 15.0	129.1 ± 21.0	125.6 ± 18.4	0.2
Creatinine	22.0 ± 3.8	24.7 ± 3.2	25.5 ± 3.2*	0.01*
Cystatin C	4.6 ± 3.7	4.8 ± 4.6	13.0 ± 10.2*	0.01*
BUN	2.6 ± 0.7	3.3 ± 0.8	3.5 ± 0.86*	0.01*
Albumin/creat. Ratio	3.0 ± 1.1	3.18 ± 2.3	2,8 ± 1.9	0.7
AST	45.7 ± 14.0	46.8 ± 10.0	40.6 ± 13.0	0.1
ALT	40.5 ± 9.0	40.0 ± 6.6	38.4 ± 9.0	0.6
Ferritin	190.8 ± 159.0	245 ± 122.0	440.0 ± 311.0**	0.001**
ESR	16.3 ± 12.0	16.7 ± 11.5	19.5 ± 11.5	0.6
CRP mg/L	4.5 ± 3.6	4.0 ± 3.57	4.0 ± 3.57	0.4

*RBC* Red blood cells, *MCV* Mean cell volume, *MCH* Mean corpuscular hemoglobin, *MCHC* Mean corpuscular hemoglobin concentration, *LDH* Lactate dehydrogenase, *WBC* White blood cell, *HDL-C* High-density lipoprotein cholesterol, *LDL-C* Low-density lipoprotein cholesterol, *AST* Aspartate aminotransferase, *ALT* Alanine amino-transferase, *CRP* C-reactive protein, *BUN* Blood Urea Nitrogen, *ESR* Erythrocyte Sedimentation Rate

**Table 6:** Association between *PON1 c.55L* > *M polymorphism* and laboratory variants

	*PON1* c.55L > M (** Χ ± SD)**			
	**LL** **(*n* = 34)**	**LM** **(*n* = 18)**	***MM*** ***(n* = *18)***	***P* value**
RBCs	3.4 ± 0.3	3.2 ± 0.4	3.0 ± 0.2*	0.01*
Haemoglobin	8.6 ± 0.9	8.4 ± 0.9	8.4 ± 0.7	0.8
Hematocrit	27.5 ± 4.7	25.6 ± 4.3	26.2 ± 4.3	0.3
MCV	87.0 ± 5.2	85.2 ± 2.6	84.0 ± 4.2	0.1
MCH	30.4 ± 3.3	29.3 ± 3.6	28.6 ± 5.5	3.36
MCHC	32.4 ± 2.7	32.1 ± 2.0	31.8 ± 1.9	0.5
Reticulocyte count	6.6 ± 3.4	9.4 ± 5.5	9.1 ± 3.0	0.08
Total bilirubin	3.0 ± 0.7	3.1 ± 0.8	3.1 ± 0.6	0.9
Direct bilirubin	0.6 ± 0.1	0.62 ± 0.1	0.6 ± 0.16	0.6
Indirect bilirubin	2.4 ± 0.6	2.5 ± 0.9	2.5 ± 0.6	0.8
LDH	492.7 ± 202	535 ± 196	592 ± 227	0.29
WBC	6.3 ± 1.1	10.4 ± 3.8	8.5 ± 3.4*	0.001**
Neutrophils	58.7 ± 6.1	61.0 ± 5.1	63.6 ± 8.0	0.13
Monocytes,	6.7 ± 2.0	7.4 ± 1.9	6.6 ± 1.3	0.34
Lymphocytes	53.7 ± 7.3	52.7 ± 5.4	49.6 ± 7.4	0.1
Platelet count	392.7 ± 99.0	372.7 ± 43	362.4 ± 82	0.56
Lipid profile				
Total Cholesterol	129.5 ± 16.0	131.0 ± 23.0	125.7 ± 18.8	0.6
HDL-C	36.5 ± 6.6	35.0 ± 4.0	36.0 ± 3.5	0.4
LDL-C	75.2 ± 13.7	69.0 ± 11.0	70.1 ± 10.0	0.19
Triglyceride	120.0 ± 21.3	118.0 ± 20.0	117.5 ± 25.0	0.5
Iron	131.0 ± 18.0	118.0 ± 15.0	122.8 ± 21.0	0.07
Creatinine	24.0 ± 3.5	23.5 ± 3.3	25.4 ± 4.0	0.25
Cystatin C	5.1 ± 3.0	5.0 ± 3.6	13.86 ± 12.2*	< 0.001**
BUN	3.0 ± 0.7	3.5 ± 0.76	3.36 ± 1.1	0.06
Albumin/creat. Ratio	2.5 ± 1.1	3.2 ± 2.1	3.76 ± 2.7	0.07
AST	45.6 ± 12.5	44.0 ± 14.2	43.3 ± 11.0	0.8
ALT	40.3 ± 8.7	40.0 ± 8.4	38.0 ± 7.9	0.65
Ferritin	236.3 ± 156.0	187.1 ± 80.0	503.0 ± 307.0	0.002*
ESR	18.5 ± 12.7	17.7 ± 10.0	15.3 ± 12.3	0.65
CRP mg/L	2.7 ± 2.2	4.3 ± 3.3	4.4 ± 3.7	0.16

*RBC* Red blood cells, *MCV* Mean cell volume, *MCH* Mean corpuscular hemoglobin, *MCHC* Mean corpuscular hemoglobin concentration, *LDH* Lactate dehydrogenase, *WBC* White blood cell, *HDL-C* High-density lipoprotein cholesterol, *LDL-C* Low-density lipoprotein cholesterol, *AST* Aspartate aminotransferase, *ALT* Alanine amino-transferase, *CRP* C-reactive protein, *BUN* Blood Urea Nitrogen, *ESR* Erythrocyte Sedimentation Rate

### Correlation between parameters and paraoxonase activity in sickle cell disease group and the control group

**Fig. 1:**
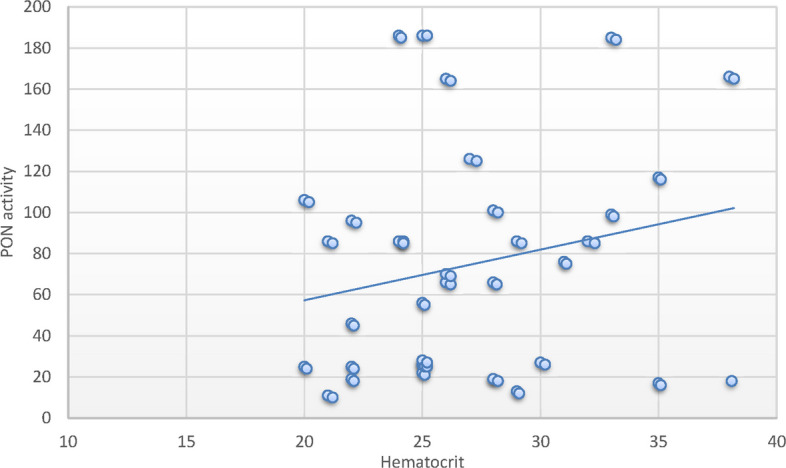
Pearson`s correlation calculated on 70 SCD patients: showed that there was a statistically significant positive correlation between PON activity and hematocrit in SCD patients (*r* = 0.26; *P* < 0.05)

**Fig. 2:**
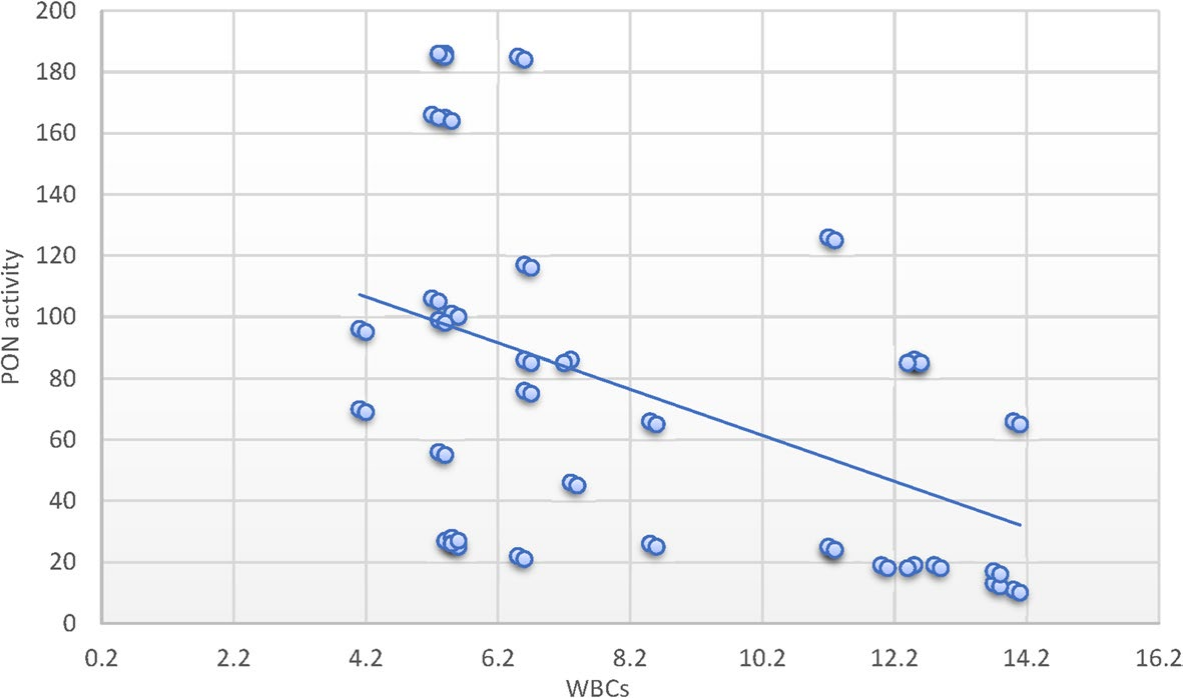
Pearson`s correlation calculated on 70 SCD patients: showed that there was a statistically significant negative correlation between PON activity and WBCs in SCD patients (*r* = −0.47; *P* < 0.001)

**Fig. 3:**
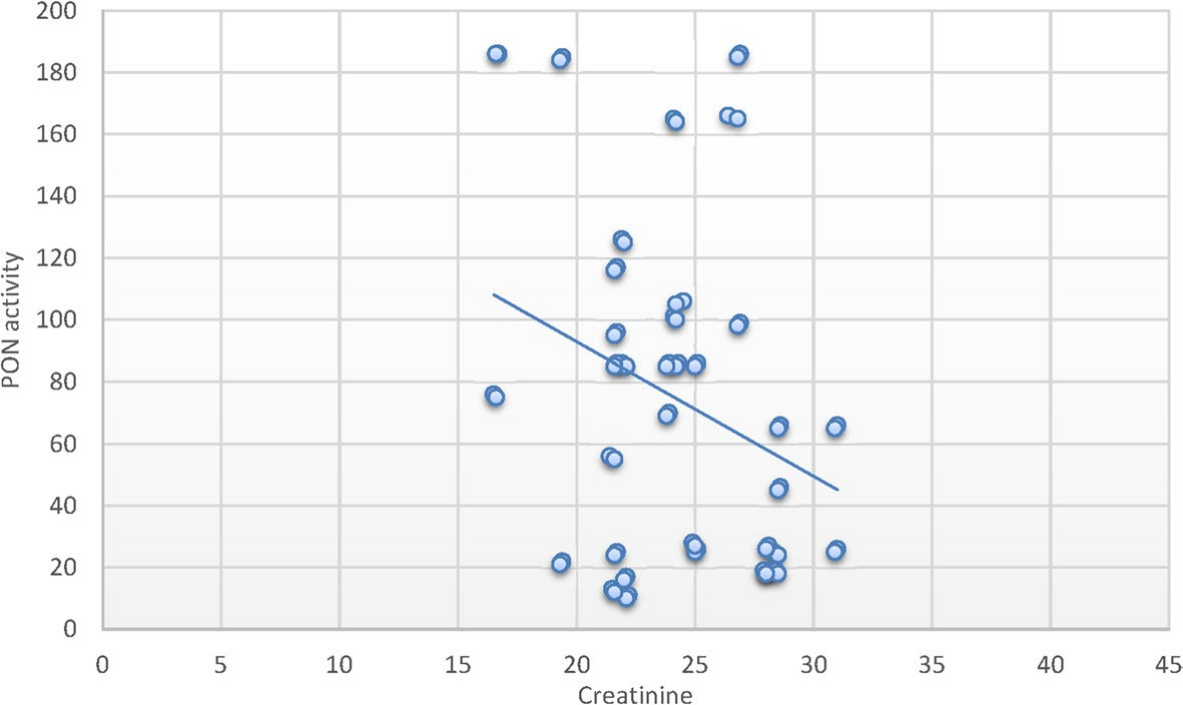
Pearson`s correlation calculated on 70 SCD patients: showed that there was a statistically significant negative correlation between PON activity and creatinine in SCD patients (*r* = − 29; *P* < 0.05)

**Fig. 4:**
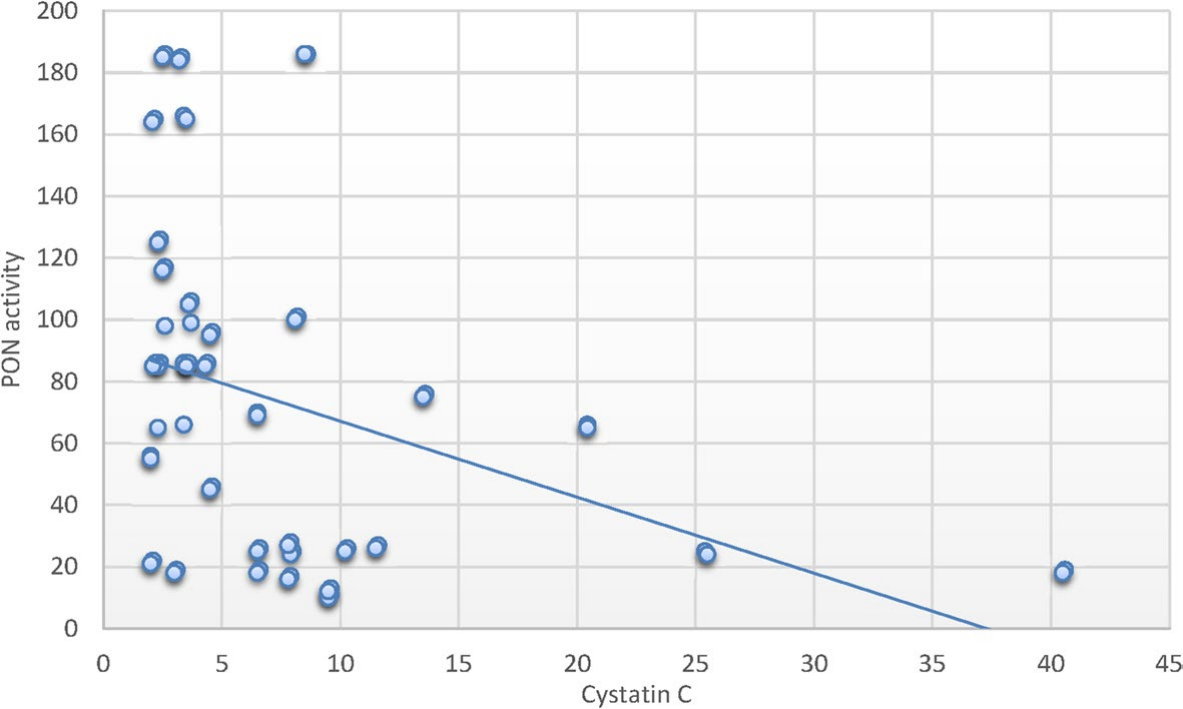
Pearson`s correlation calculated on 70 SCD patients: showed that there was a statistically significant negative correlation between PON activity and cystatin C in SCD patients (*r* = − 37; *P* < 0.001)

**Fig. 5:**
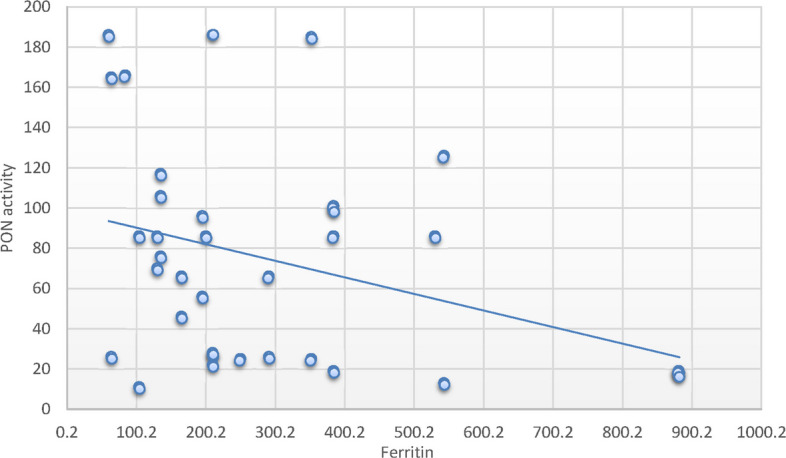
Pearson`s correlation calculated on 70 SCD patients: showed that there was a statistically significant negative correlation between PON activity and ferritin in SCD patients (*r* = − 0.34; *P* < 0.05

A positive correlation between hematocrit and PON-1 activity (*r* = 0.26; *P* < 0.05) (Fig. 1) was identified in SCD patients. Conversely, negative correlations were found with WBCs (*r* = −0.47; *P* < 0.001) (Fig. 2), creatinine (*r* = − 29; *P* < 0.05) (Fig. 3), cystatin C (*r* = − 37; *P* < 0.001) (Fig. 4), and ferritin levels (*r* = − 0.34; *P* < 0.05) (Fig. 5), while other parameters showed no significant correlation as demonstrated in supplementary 4. controversary there was no significant correlation between paraoxonase activity and same parameters in control group, this distinction improves the interpretation of PON1’s role in SCD pathophysiology (supplementary 5).

## Discussion

We detected a statistically significant reduced PON-1 expression and lower levels of enzymatic activity in patients with SCD compared to controls. In line with these findings, El-Ghamrawy et al. reported significantly lower PON levels in Egyptian children with SCD [18]. Reichert et al. detected reduced PON 1 activities in the SCD group compared with controls. However, it did not reach statistical significance [19].

In contrast, Menezes et al. detected increased PON 1 activity in SCD patients compared to the control group [14]. Numerous variables could be contributing to the discrepancy between the studies, including patient demographics, sample size variations, and methodologies.

SCD is increasingly recognized as a significant oxidative stress model arising from the imbalance between antioxidant enzymes and reactive oxygen species (ROS), leading to endothelial injury, chronic inflammation, and multiple organ damage [18, 20]. Moreover, oxidative stress has been reported due to increased free iron levels in diseases with iron overload [21]. PON-1 circulates in the plasma bound to HDL and can be transported to tissues to exert its crucial antioxidant role [22, 23]. The oxidation of HDL structure and the reduction in its PON-1 content have been documented during chronic inflammation [21]. The observed findings of diminished PON-1 expression and enzymatic activity in SCD patients may be elucidated by these findings.

In our study, the genotype distribution for PON1 c.55L > M among controls conformed to Hardy–Weinberg equilibrium (HWE), indicating no significant deviation and supporting the validity of the genotyping process. However, a slight deviation from HWE was noted for the PON1 c.192Q > R polymorphism in the control group (*p* = 0.035). This deviation may reflect random sampling variation, population stratification, or technical artifacts. Although modest, it should be interpreted with caution, and further validation in larger and more genetically diverse control cohorts is recommended to confirm the stability of this polymorphism.

We observed a significantly higher frequency of the PON1c.192R allele in the SCD group than in the control group. Both QR and RR genotypes were significantly associated with an increased risk of SCD when compared to the QQ genotype, suggesting a potential role of this variant in disease susceptibilitly.

Alternatively, the *PON1*c.55L > M *variant allele* had no significant association with SCD risk.

Higher frequencies of RR and QR and lower frequencies of QQ in our SCD group were unexpected compared to healthy controls. These findings partially support the findings of Reichert et al., who reported a higher frequency of the RR and a low frequency of the QQ genotype in patients with SCD [19]. In contrast, Menezes et al. identified higher allelic frequencies of the PON1c.192Q and PON1c.55L [14]. These differences reflect the population-specific genetic variability.

A previous study identified QQ, QR, and RR genotypes in 12%, 43%, and 45% of Caucasians from Europe and America, respectively, also reported a higher prevalence of the L allele in Chinese and Japanese (91–94%) as well as Caucasians (67–74%) [24]. Another research in Brazil documented more elevated R and L allele frequencies (97% and 73%, respectively) [25]. This discrepancy may be linked to ethnic variations in the PON-1 SNP genotype and the regulation of its expression. The variation in genotype distribution of PON-1 among Egyptians compared to other populations may be associated with the genetic diversity within the Egyptian population; Egyptians are regarded as a mixed-race population [26].

Previous studies have suggested that PON-1 polymorphisms might influence the enzymatic activity [8, 19, 25]. In the current study, SCD subjects with the homozygote variant *PON1*c.192 RR, *PON1*c.55 LL and MM genotypes exhibited decreased PON1 activity compared with control subjects with identical genotypes. Consistent with these findings, Menezes et al. found that the variant PON1c.192R correlated with downregulated enzyme activity. However, they reported elevated PON1 activity in the PON1c.55LL and PON1c.192QR genotypes. Variant (LM + MM) genotype carriers exhibited minimized PON1 activity compared to individuals with the LL genotype. Similarly, individuals with the QR + RR genotype exhibited minimized PON1 activity in comparison to the QQ genotype [14].

Conversely, Reichert et al. documented higher PON activity with the PON1c.192RR genotype in patients with SCD. The PON1c.55LM variant was associated with diminished PON1 activity compared with control subjects possessing the corresponding genotype [19].

The findings indicate that the PON1 gene variant alleles may pose potential risks for patients with SCD. Further studies are necessary to confirm this, considering the population's genetic diversity.

We found that SCD patients with RR genotype had lower disease duration and increased levels of WBCs, LDH, ferritin, creatinine, cystatin C, and BUN, alongside reduced RBC indices. In contrast, patients carrying the QQ genotypes were substantially more likely to have higher HbSS, ACS < 1, and receive no chelation therapy.

Concerning *PON1* c.55L > M, we discovered marked correlations between the variant MM genotype, lower RBCs, WBC count, serum ferritin, and cystatin C levels. The observed outcomes may be attributed to oxidative stress associated with the diminished PON1 activity in this population. Oxidative stress exacerbates RBC sickling, increasing hemolysis and inflammation [27]. Elevated LDH indicate intravascular hemolysis, vascular obstruction, and endothelial dysfunction [1, 28]. In patients with SCD, increased serum ferritin correlated with reactive oxygen species production, iron overload, and multiple organ dysfunctions [29].

Elevated creatinine, cystatin C, and BUN levels in the RR genotype and elevated cystatin C levels in the MM genotype may be linked to the renal and cardiovascular dysfunction associated with PON antioxidant dysfunction. Cystatin C is considered a reliable marker of renal function, particularly in mild renal impairment. Moreover, it exhibited a correlation with an elevated cardiovascular dysfunction risk in SCD patients [30]. Our results align with studies linking the R genotype and diminished PON-1 activity to complications in other conditions, such as chronic kidney disease and cardiovascular dysfunction, further emphasizing thier clinical significance [31–33].

In contrast to our findings, Kotani et al. indicated that the RR genotype may serve as a protective factor against oxidative stress, evidenced by elevated PON activities and diminished serum ferritin levels in SCD patients [34], aligning with Reichert et al. [19]. These discrepancies may result from ethnic, environmental, or methodological differences.

PON1 plays a key role in lipid metabolism by protecting LDL from oxidation, supporting HDL function, and preventing atherosclerosis [35–37]. However, our study found no significant association between PON1 polymorphisms and lipid profile. In line with our data, Menzies et al. found no significant association between the investigated polymorphisms and HDL levels. Nevertheless, lower VLDL-c and triglyceride levels were detected in SCD patients with the variant (QR + RR) genotype [14]. Conversely, Reichert et al. detect a positive correlation between HDL-C levels and PON1 activity in SCD patients [19].

Correlation analyses between laboratory variables and PON1 activity in patients with SCD revealed a significant positive correlation with hematocrit and a significant negative correlation with WBCs, creatinine, cystatin C, and serum ferritin levels. These findings corroborate the aforementioned association analysis concerning the role of PON1 in organ injury linked to diminished activity and compromised antioxidant capacity in SCD patients. These findings have potential clinical relevance. Reduced PON1 activity and its association with adverse renal markers such as cystatin C and elevated BUN may serve as early indicators of renal dysfunction in pediatric SCD patients. Given the role of oxidative stress in SCD pathophysiology, identifying patients with risk-associated PON1 genotypes could enable early risk stratification. Moreover, these insights may open new avenues for personalized interventions, such as antioxidant therapy or dietary modifications aimed at enhancing PON1 activity, to prevent or delay cardiovascular and renal complications in children with SCD.

## Conclusion

*PON1*c.55L > M as well as *PON1*c.192Q > R gene polymorphisms in SCD patients, displayed differences when compared to controls. We found that genotype QR and RR were significantly associated with increased SCD risk. In the current study, SCD patients with the variant *PON1*c.192 RR, *PON1*c.55 LL and MM genotypes exhibited decreased PON1 activity. Decreased PON-1 activity in SCD patients was associated with worsening hemolysis and inflammation, higher ferritin, and elevated creatinine, cystatin C, and BUN levels. This may predict future detrimental clinical outcomes, along with potential risk for renal and cardiovascular impairment. Additional research on a larger sample is necessary to validate the PON-1 gene polymorphism as a risk factor for patients with SCD and to gain a comprehensive understanding of the translational regulation of PON-1.

### Limitations

This study has several limitations that should be acknowledged. First, the relatively small sample size may limit the generalizability and statistical power, particularly within genotype subgroup analyses. Second, the study population was limited to Egyptian children and adolescents, and the findings may not be applicable to other ethnic or geographic populations due to potential genetic and environmental variability in PON1 polymorphisms and expression. Additionally, while we controlled for key confounders such as hydroxyurea use and transfusion history, unmeasured factors such as nutritional status, oxidative stress from infections, or other genetic modifiers may still influence PON1 activity. Future multicenter studies with larger, ethnically diverse cohorts and longitudinal follow-up are warranted to validate and expand upon these findings.

## Supplementary Information

Supplementary Material 1.

## Data Availability

No datasets were generated or analysed during the current study.
