# Supplementary material for: Association of PON1 gene polymorphisms, paraoxonase 1 gene expression and activity in Egyptian children and adolescents with sickle cell disease
Source: BMC Pediatr. 2026 Mar 21;26:284. doi: 10.1186/s12887-026-06597-w (PMC13063545; doi:10.1186/s12887-026-06597-w)
Supplement: Supplementary file 1 — Supplementary Material 1. [file 12887_2026_6597_MOESM1_ESM.docx]

**Supplementary 1: Associations between 2 SNPs in selected ATG genes with breast cancer risk.**

| SNP | Allele ^a^ | Case ^b^, N = 70 | Control ^b^, N = 70 | MAF ^c^ (case/control) | HWE ^d^  case/control | OR (95%CI) | *P* |
| --- | --- | --- | --- | --- | --- | --- | --- |
| *PON1 c.192Q > R* | Q/R | 18/30/22 | 42/20/8 | 0.528/0.257 | 0.24/0.035 | 6.4 (2.19-19.4) | 0.001* |
| *PON1*c.55L > M | L/M | 34/18/18 | 32 /26/12 | 0.457/0.357 | <0.001*/0.11 | \ | 0.2 |

a Major/minor allele;

b Major homozygote/heterozygote/rare homozygote between cases and controls;

c Minor allele frequency;

d Hardy–Weinberg equilibrium test among case/controls;

**Supplementary 2: Comparison between** **SCD patients and controls regarding *PON1* c.55L > M Genotype and allele frequency**

| **Polymorphism** | **SCD** | | **Control** | | | | ***OR (95%CI)*** | |  |
| --- | --- | --- | --- | --- | --- | --- | --- | --- | --- |
|  | **N** | **%** | | **N** | | **%** | |  | |
| ***PON1*c.55L > M** | | | | | | | | |  |
| LL | 34 | 48.6 | 32 | | 45.7 | | 1 | |  |
| LM | 18 | 25.7 | 26 | | 37.1 | | 0.65(0.28-1.5) | |  |
| MM | 18 | 25.7 | 12 | | 17.1 | | 1.41(0.54-3.7) | |  |
| Allele frequency | | | | | | | | |  |
| L | 86 | 61.4 | 90 | | 64.3 | | 1 | |  |
| M | 54 | 38.6 | 50 | | 35.7 | | 1.13(0.68-1.09) | |  |

**Supplementary 3: Association between PON1 c.55L > M polymorphism and demographic and clinical variants:**

|  | ***PON1* c.55L > M** | | | | | | |
| --- | --- | --- | --- | --- | --- | --- | --- |
| **Variant** | LL | | LM | | *MM* | | *P* |
|  | N= 34 | % | N= 18 | % | N=18 | % |  |
| Sex  Male  Female | 16  18 | 47.1  52.9 | 14  4 | 77.8  22.2 | 10  8 | 55.6  44.4 | 0.1 |
| Age (Χ±SD) | 9.00±4.00 | | 10.1±2.5 | | 7.5±3.1 | | 0.08 |
| Duration (Χ±SD) | 6.7±4.00 | | 7.6±2.7 | | 4.8±3.3 | | 0.06 |
| Family history  -ve  +ve | 10  24 | 29.4  70.6 | 4  14 | 22.2  77.8 | 6  12 | 33.3  66.7 | 0.75 |
| Diagnosis  HbSS  HbSB  HbSC | 22  10  2 | 64.7  29.4  5.9 | 8  8  2 | 44.4  44.4  11.1 | 12  6  0 | 66.7  33.3  0.0 | 0.44 |
| VOC  ≤ 1  ≥ 2 | 14  20 | 41.2  58.8 | 8  10 | 44.4  55.6 | 12  6 | 66.7  33.3 | 0.19 |
| ACS  ≤ 1  ≥ 2 | 30  4 | 89.2  11.8 | 14  4 | 77.8  22.2 | 16  2 | 88.9  11.1 | 0.5 |
| Transfusion  No  Occasional | 12  22 | 35.3  64.7 | 4  14 | 22.2  77.8 | 8  10 | 44.4  55.6 | 0.36 |
| Chelation  No  Yes | 28  6 | 82.4  17.6 | 14  4 | 77.8  22.2 | 16  2 | 88.9  11.2 | 0.63 |

**Supplementary 4: Correlation between parameters and paraoxonase activity in sickle cell disease group**

| **Laboratory parameters** | **R** | ***P* value** |
| --- | --- | --- |
| Hemolysis markers | | |
| Total bilirubin, | 0.02 | NS |
| Direct bilirubin, | 0.18 | NS |
| Indirect bilirubin, | 0.05 | NS |
| LDH | 0.05 | NS |
| Hemoglobin | -0.19 | NS |
| Hematocrit | 0.26 | <0.05* |
| Leukocytes | | |
| Reticulocyte | 0.01 | NS |
| WBC | -0.47 | <0.001** |
| Iron metabolism | | |
| Iron | -0.05 | NS |
| Renal profile | | |
| Creatinine | -0.29 | <0.05* |
| Urea | -0.12 | NS |
| Cystatin C | -0.37 | <0.001** |
| Albumin/creat. ratio | -0.07 | NS |
| Hepatic profile | | |
| AST, U/L | 0.08 | NS |
| ALT, U/L | 0.2 | NS |
| Inflammatory biomarkers | | |
| Ferritin, ng/dL | -0.34 | <0.05* |

**Supplementary 5: Correlation between parameters and paraoxonase activity in control group**

| **Laboratory parameters** | **R** | ***P* value** |
| --- | --- | --- |
| Hemolysis markers | | |
| Total bilirubin, | 0.04 | NS |
| Direct bilirubin, | 0.08 | NS |
| Indirect bilirubin, | 0.07 | NS |
| LDH | 0.09 | NS |
| Hemoglobin | -0.15 | NS |
| Hematocrit | 0.03 | NS |
| Leukocytes | | |
| Reticulocyte | 0.018 | NS |
| WBC | -0.03 | NS |
| Iron metabolism | | |
| Iron | 0.2 | NS |
| Renal profile | | |
| Creatinine | -0.14 | NS |
| Urea | -0.17 | NS |
| Cystatin C | -0.08 | NS |
| Albumin/creat. ratio | -0.06 | NS |
| Hepatic profile | | |
| AST, U/L | 0.02 | NS |
| ALT, U/L | 0.17 | NS |
| Inflammatory biomarkers | | |
| Ferritin, ng/dL | -0.01 | NS |
